# Supplementary material for: Transglutaminase 2 Facilitates Murine Wound Healing in a Strain-Dependent Manner
Source: Int J Mol Sci. 2023 Jul 14;24(14):11475. doi: 10.3390/ijms241411475 (PMC10380275; doi:10.3390/ijms241411475)
Supplement: Supplementary file 1 [file ijms-24-11475-s001.zip › ijms-2506554-supplementary.pdf]

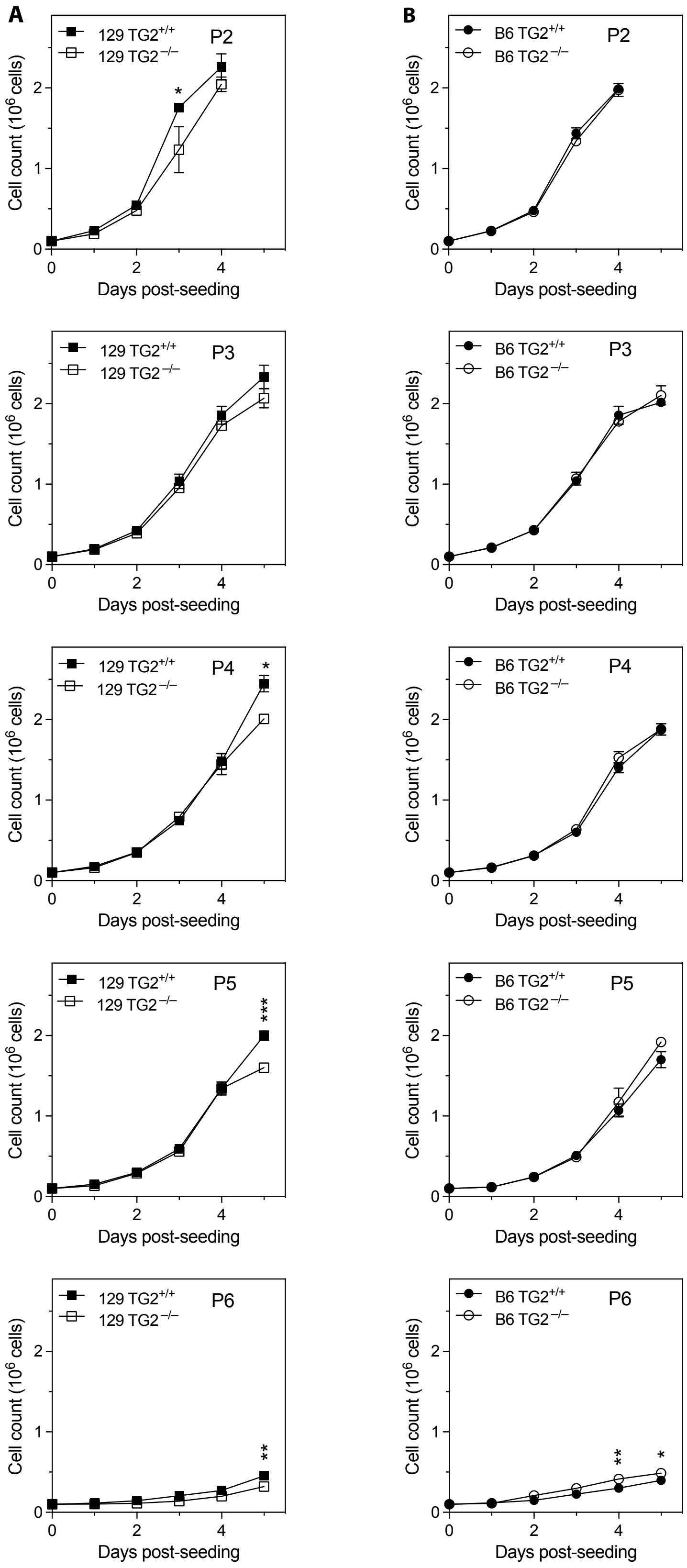

Figure S1. MEF proliferation was drastically reduced with increasing passages. Random batches of (**A**) 129 and (**B**) B6 TG2$^{+/+}$ and TG2$^{-/-}$ MEFs (*n* = 3) were monitored for growth rate at passage 2 (P2), P3, P4, P5 and P6. At P2, cells reached confluence on day 4 and for subsequent passages, up to P5, confluence was reached on day 5. Cells were split on the day they reached confluency. MEFs did not propagate beyond P6. *, $p < 0.05$; **, $p < 0.01$, ***, $p < 0.001$ for two-way ANOVA with post hoc Bonferroni correction.

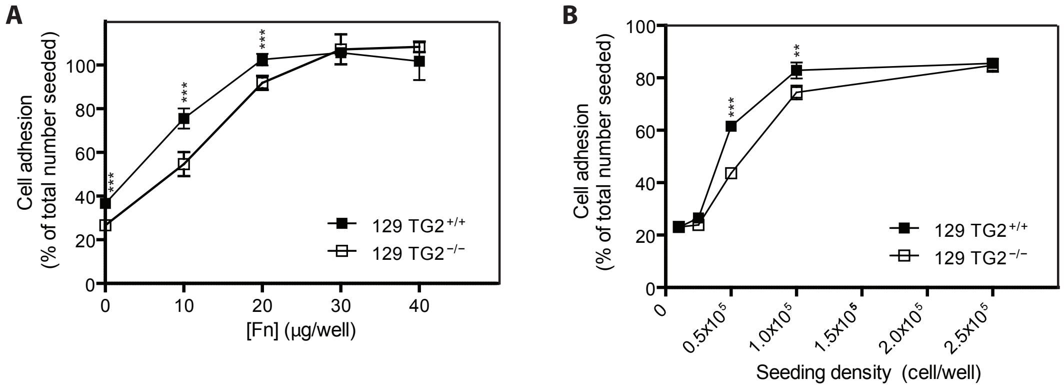

Figure S2. Effect of fibronectin (Fn) concentration or seeding density on adhesion of 129 TG2$^{+/+}$ and 129 TG2$^{-/-}$ MEFs. (**A**) Fifty thousand 129 TG2$^{+/+}$ or TG2$^{-/-}$ MEFs were seeded onto wells of a 24-well plate that were pre-coated with 0 to 40 µg Fn/cm^2 and adherent cells were quantitated after 30 min (n = 3 experiments, in triplicate). (**B**) 129 TG2$^{+/+}$ or TG2$^{-/-}$ MEFs were seeded at increasing density onto wells of a 24-well plate pre-coated with 10 µg Fn/cm^2 and adherent cells were quantitated after 30 min (n = 3 experiments performed in triplicate). The greatest difference in adhesion of 129 TG2$^{+/+}$ and TG2$^{-/-}$ MEFs was observed with a Fn concentration of 10 µg/cm^2 and a cell density of 5x10^4/cm^2. **, $p < 0.01$; ***, $p < 0.001$ for two-way ANOVA with post hoc Bonferroni correction.

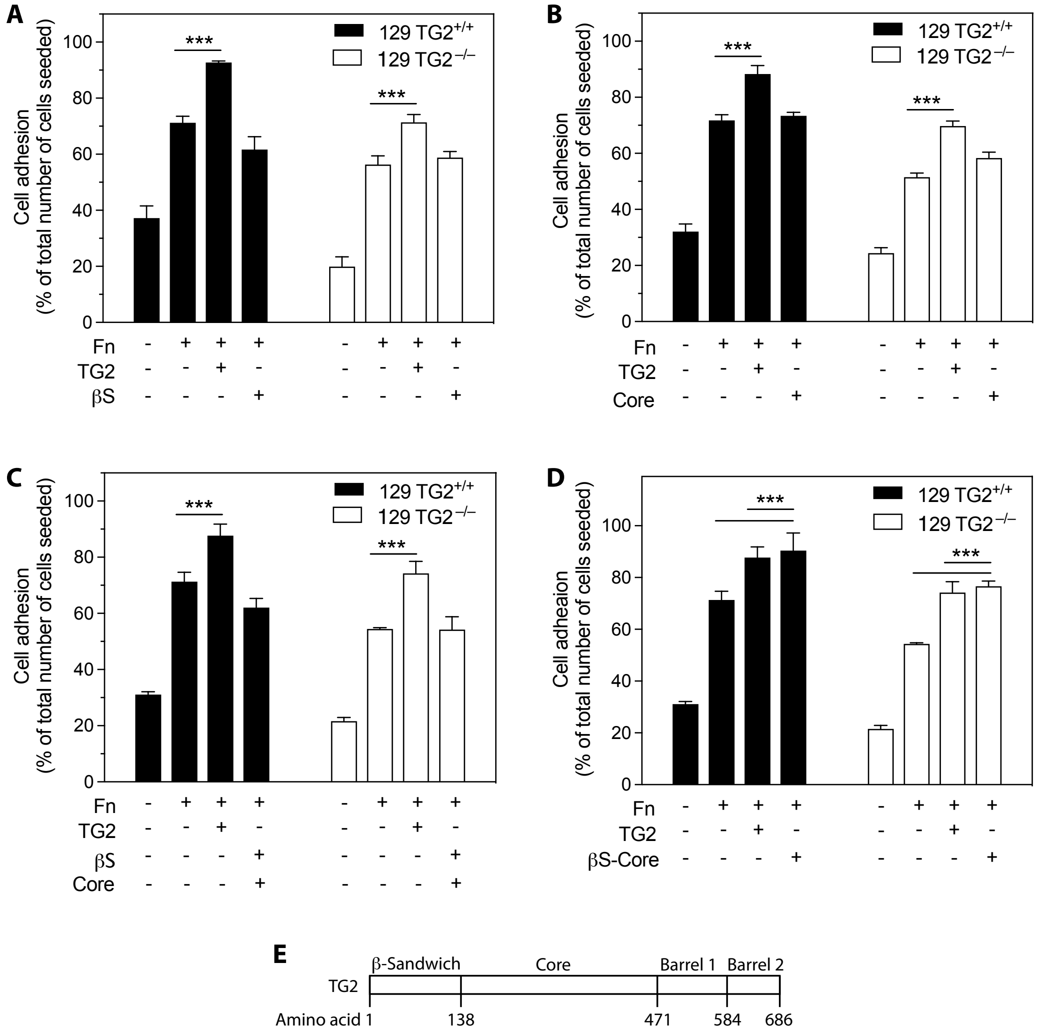

Figure S3. The β-sandwich-core domain of TG2, but not the β-sandwich or core domain alone, was able to substitute for full-length TG2 to increase the number of adherent 129 TG2$^{+/+}$ and 129 TG2$^{-/-}$ MEFs. (**A, B, C, D**) Quantitation of adherent 129 TG2$^{+/+}$ or 129 TG2$^{-/-}$ MEFs after 30 min incubation on plates coated without Fn, with Fn alone, with Fn plus full-length TG2 (20 μg/cm^2), or with Fn plus equivalent molar amounts of (A) β-sandwich domain of TG2 (amino acids 1-138, [59]) (n = 3 experiments performed in triplicate), (B) core domain of TG2 (amino acids 139-471, [59]) (n = 3 experiments performed in triplicate), (C) β-sandwich and core domains of TG2 (n = 4 experiments performed in triplicate) or (D) β-sandwich-core domain of TG2 (amino acids 1-471, [59]) (n = 4 experiments performed in triplicate), expressed as a percentage of the total number of cells (5x10^4) seeded. (**E**) Diagrammatic representation of the domain structure of full-length TG2. ***, $p < 0.001$ for a two tailed Student t-test.

Table S1. Genes with mRNA abundance changes of at least 0.1 log-fold ($p < 0.05$) from microarray analysis of total RNA from 129 TG2$^{-/-}$ and 129 TG2$^{+/+}$ skin wounds.

Gene symbol	log-fold change	p.value
Tgm2	-2.25266	3.61E-08
Mup2	-0.99513	0.044244
Rrad	-0.94954	0.002651
Klhl31	-0.89374	0.028146
Apobec2	-0.86925	0.008187
Xirp2	-0.85486	0.029905
Smpx	-0.84001	0.008905
Kbtbd10	-0.83936	0.014641
Myoz2	-0.76154	0.028233
Asb5	-0.71936	0.035307
Nrap	-0.71351	0.0372
Lrrc2	-0.69317	0.032042
Des	-0.68684	0.024143
Tnnc2	-0.6663	0.042856
Tnni2	-0.62209	0.046102
Mb	-0.61904	0.030163
Srl	-0.61187	0.032999
Mustn1	-0.58975	0.012266
Pkia	-0.58651	0.031822
Mylk2	-0.58112	0.037744
Mmp13	-0.55315	0.023628
Usp13	-0.54959	0.018649
EG382931	-0.54584	0.004871
Acta1	-0.54158	0.040607
Tspan8	-0.53317	0.043318
EG667441	-0.52767	0.003546
100039528	-0.5065	0.010237
Gadl1	-0.48945	0.027436
Eef1a2	-0.48938	0.030035
Asb14	-0.48806	0.028284
Rragd	-0.47859	0.016296
Cox6a2	-0.46071	0.038904
OTTMUSG00000017746	-0.45821	0.001451
Tas2r135	-0.45336	0.006623
Cryab	-0.45185	0.044414
Prkg1	-0.44182	0.048171
Trim72	-0.44149	0.022915
Lmod2	-0.43139	0.036902
Olfr1148	-0.41777	0.002768
Myo18b	-0.41563	0.039337
AA416372	-0.41311	0.011337
LOC100045610	-0.41165	0.025075
Serpinb1a	-0.40274	0.031873
Dusp27	-0.3998	0.025337
Myo18b	-0.39751	0.010706

OTTMUSG00000007808	-0.37206	0.022392
ENSMUSG00000074845	-0.3579	0.004762
Cnksr1	-0.35501	0.036583
Kcnc4	-0.35324	0.006386
Ptgs2	-0.35217	0.047123
Khdc1a	-0.34369	0.002499
Unc45b	-0.3436	0.04265
Lrrn1	-0.3406	0.045767
Tgm7	-0.33956	0.015476
Smtnl1	-0.3395	0.003997
Olfr310	-0.33731	0.002365
OTTMUSG00000007474	-0.33113	0.032497
Tas2r143	-0.32702	0.016844
Myo18b	-0.32498	0.042229
Fbxo40	-0.32312	0.009443
Atp1b1	-0.32139	0.012909
Kcna7	-0.32006	0.002281
Sync	-0.31903	0.041394
Olfr373	-0.31845	0.025584
Kcnq5	-0.31399	0.032714
Apcs	-0.31358	0.009282
Krtap5-1	-0.31254	0.002391
Musk	-0.30698	0.03447
Abcb4	-0.30549	0.020088
Ihpk3	-0.30523	0.025864
Olfr605	-0.30324	0.026712
Klk1b24	-0.30161	0.022554
C1qtnf3	-0.29754	0.005602
Ak1	-0.29567	0.020939
Olfr239	-0.29538	0.005394
Sh3bgr	-0.29536	0.038447
F2rl3	-0.28792	0.003993
Cfl2	-0.28769	0.045883
ENSMUSG00000073569	-0.28152	0.010332
Gm996	-0.27902	0.021061
EG218444	-0.27871	0.029302
Kir3dl1	-0.27604	0.032181
Synpo2l	-0.27513	0.037758
Ociad2	-0.26812	0.017785
ENSMUSG00000072690	-0.26491	0.019088
Olfr1031	-0.2636	0.010467
OTTMUSG00000008822	-0.25881	0.001785
LOC627626	-0.25802	0.04568
Vmn2r87	-0.25161	0.032898
EG620119	-0.25148	0.038811
Olfr957	-0.24796	0.024398
Avpr2	-0.24754	0.008686
A130082M07Rik	-0.24699	0.032031
Crhr2	-0.24566	0.01745

Dnajc5b	-0.24429	0.04422
Srms	-0.24408	0.004572
Sema6c	-0.24207	0.010198
Hormad1	-0.24167	0.01298
Rprm	-0.24065	0.018237
LOC676914	-0.24041	0.035641
Mkrn3	-0.24009	0.002989
Shisa2	-0.23957	0.031507
Mmp10	-0.23896	0.032533
Stac3	-0.23867	0.017624
OTTMUSG00000005131	-0.2384	0.016192
Gpr25	-0.23838	0.005103
100040113	-0.238	0.005613
LOC100048255	-0.2379	0.026365
Myh6	-0.23537	0.017544
EG628893	-0.23427	0.020705
Olfr1364	-0.23272	0.012392
Itgb1bp3	-0.23242	0.044022
Lmbr1	-0.23241	0.041136
6430517E21Rik	-0.2322	0.000818
Kcnj11	-0.23219	0.010002
Lrrtm3	-0.23208	0.015281
Speer2	-0.23139	0.021965
Neurl	-0.23124	0.038216
Sema4g	-0.23079	0.004594
ENSMUSG00000074555	-0.22825	0.014433
Cyp2d10	-0.22825	0.016883
ENSMUSG00000074072	-0.22796	0.008609
Igk-V1	-0.22514	0.047424
EG666123	-0.22489	0.036902
Tgfb2	-0.22473	0.021239
Mpo	-0.22439	0.047303
1110030E23Rik	-0.22397	0.042011
Slco5a1	-0.22337	0.024639
Olfr350	-0.22327	0.037706
Hspb2	-0.22282	0.046337
Tas2r129	-0.22235	0.02447
9430060I03Rik	-0.22189	0.019093
Htr5b	-0.22179	0.005622
EG546708	-0.22176	0.017049
Tnnt2	-0.22158	0.036705
Kcnj5	-0.22149	0.019185
621968	-0.2203	0.017832
EG624023	-0.22	0.048371
ENSMUSG00000071392	-0.21964	0.017485
A430072C10Rik	-0.21912	0.02293
LOC622659	-0.21763	0.02644
Atp8b3	-0.21726	0.035614
B4galnt2	-0.21711	0.038741

Syt10	-0.21669	0.008492
Il28a	-0.21598	0.041862
Hspb3	-0.21593	0.044028
Slc23a1	-0.21577	0.009136
V1rd15	-0.21545	0.04634
EG668156	-0.21545	0.037941
Fgf20	-0.21528	0.010173
Olfr464	-0.2139	0.04477
Nlrp14	-0.21383	0.013414
Sox8	-0.21327	0.011115
4930471G03Rik	-0.21295	0.015523
Olfr557	-0.21231	0.019395
Ovol3	-0.21086	0.042808
V1rg6	-0.21053	0.039398
Ctsg	-0.21018	0.031171
Taar5	-0.21009	0.032926
Isl1	-0.20826	0.01206
V1rg8	-0.20754	0.039637
Nefl	-0.20715	0.010202
Camp	-0.20697	0.023225
Glyctk	-0.20654	0.032992
Skap1	-0.20633	0.021563
H2-M10.2	-0.20491	0.012051
Insm2	-0.20431	0.018205
Col4a6	-0.20428	0.012816
Tgif2	-0.20409	0.035834
Ppifos	-0.20385	0.005462
Tff2	-0.20356	0.010665
Tmem139	-0.20348	0.029328
Rdh19	-0.20301	0.012137
Pnma3	-0.20294	0.008392
Gpr158	-0.20282	0.010118
EG382156	-0.2027	0.042222
D430041D05Rik	-0.20182	0.019249
A930035D04Rik	-0.20025	0.039357
2610021K21Rik	-0.20007	0.032767
Pdc	-0.19977	0.015245
Slc13a4	-0.19975	0.034007
9530053H05Rik	-0.19967	0.018619
1700013G24Rik	-0.19941	0.016036
OTTMUSG00000023126	-0.19927	0.04429
1110001D15Rik	-0.19908	0.034463
LOC546695	-0.19805	0.043672
Adamts3	-0.19781	0.039381
Cnih3	-0.19755	0.041114
3322402L07Rik	-0.19744	0.020407
Megf6	-0.19718	0.038448
LOC100044855	-0.19691	0.006297
Nhedc2	-0.19635	0.025083

Wdr16	-0.19615	0.007324
4921530L21Rik	-0.196	0.026632
Olfr1272	-0.1959	0.039068
Tram1l1	-0.19529	0.015511
LOC100045615	-0.19484	0.029621
Kif12	-0.19365	0.008537
Mycs	-0.19298	0.032547
Prhoxnb	-0.19294	0.0344
Gast	-0.19274	0.016107
V1ra2	-0.1926	0.041092
Hsf2	-0.19221	0.039006
Kcnh7	-0.1918	0.02181
Pcsk9	-0.19055	0.020233
Olfr611	-0.19046	0.035995
Ctsr	-0.19029	0.009526
4921509A18Rik	-0.19007	0.009605
Dab1	-0.18971	0.031283
EG639530	-0.18961	0.011469
Slc6a15	-0.18943	0.038577
Lefty2	-0.18915	0.039255
Cd209e	-0.18902	0.035945
Egfl6	-0.18884	0.023113
Rit2	-0.1873	0.038794
Olfr1511	-0.18713	0.035411
Gm568	-0.18684	0.019182
Glis1	-0.18679	0.0267
Amhr2	-0.18668	0.013659
Wdr31	-0.18623	0.016842
Cacna1g	-0.18563	0.039921
Crygn	-0.18554	0.01444
Ica1l	-0.18553	0.007614
Plb1	-0.18552	0.029057
Vgll1	-0.1855	0.022826
Tcp11	-0.18545	0.014324
Pga5	-0.18539	0.017111
Sh3rf3	-0.18413	0.006472
Slc12a5	-0.18403	0.023018
Olfr180	-0.1836	0.01275
Chrna9	-0.18355	0.042787
Gpr20	-0.18354	0.044477
Col9a3	-0.18292	0.020421
EG218997	-0.1823	0.042421
Olfr390	-0.18181	0.046352
Apol8	-0.18176	0.02744
Sstr2	-0.18175	0.029653
Sphkap	-0.18147	0.014015
Zfp651	-0.1814	0.029486
Prf1	-0.18136	0.028853
Olfr1305	-0.18133	0.040487

Nr2e3	-0.18084	0.013504
Dcakd	-0.18079	0.019358
A4gnt	-0.18067	0.009931
Stmn3	-0.18059	0.016294
Dnajc6	-0.18034	0.023619
Zfp264	-0.18011	0.01628
Cntnap5c	-0.17983	0.021066
Mos	-0.17924	0.016024
Banf2	-0.17861	0.010025
2810051F02Rik	-0.17849	0.049897
Igf2as	-0.17833	0.011878
Ern2	-0.17748	0.021993
Cryba4	-0.17684	0.038092
Cst11	-0.17671	0.042793
Slc6a3	-0.17656	0.018306
Ina	-0.17641	0.032588
Olfr1231	-0.1762	0.044051
Nlgn1	-0.1759	0.021283
EG330305	-0.17566	0.036242
Il27	-0.17562	0.038789
Vax2	-0.17526	0.014942
2900005J15Rik	-0.17507	0.041133
Myo18b	-0.1738	0.015721
Olfr157	-0.1732	0.033746
Tchhl1	-0.17295	0.033893
Rhcg	-0.17188	0.022235
Lrrc50	-0.17176	0.047765
Slc25a29	-0.17147	0.026488
Bmp8b	-0.17092	0.032086
Prdm13	-0.17076	0.018772
1600012P17Rik	-0.17036	0.019166
Lrfn1	-0.1701	0.046124
EG629583	-0.17007	0.033131
Impg2	-0.16989	0.0315
Mug1	-0.16977	0.025881
Ppm1e	-0.16964	0.015225
Aqp12	-0.16946	0.018625
1700018M17Rik	-0.16859	0.03948
Htr2b	-0.16841	0.040263
ENSMUSG00000072619	-0.16716	0.047356
Clec4a4	-0.16655	0.015616
Tmem91	-0.1663	0.016442
Ank1	-0.16568	0.018855
Serpina5	-0.16544	0.047745
Kifc5c	-0.16531	0.011217
Fgf12	-0.16442	0.023106
Slc1a4	-0.16403	0.027653
Otos	-0.16399	0.03192
Fstl4	-0.16383	0.02757

Ak5	-0.16355	0.046123
Gpr3	-0.16352	0.031405
Spink4	-0.16272	0.047458
Rbm11	-0.16256	0.048175
Adra2c	-0.16253	0.033032
Slc13a5	-0.16207	0.007239
EG546638	-0.16161	0.035673
Gm1323	-0.16143	0.031838
2810055G20Rik	-0.16082	0.049596
Dyx1c1	-0.16031	0.013626
Olfr1030	-0.16031	0.037131
Phxr1	-0.16016	0.03811
Ccdc136	-0.16002	0.014545
Nkx6-1	-0.16001	0.038953
Ncf2	-0.15962	0.049651
Onecut1	-0.15961	0.049195
D930048N14Rik	-0.15922	0.047464
Abcc8	-0.15775	0.034015
Traf4	-0.15747	0.039724
Stmn4	-0.15637	0.024327
4930550C14Rik	-0.15611	0.042949
Cd276	-0.15584	0.038204
Grm8	-0.15563	0.030321
ENSMUSG00000052323	-0.15496	0.030708
Rhox6	-0.15493	0.029318
Acpt	-0.15452	0.043986
Artn	-0.15373	0.01941
Olfr1038	-0.15273	0.031516
Slc17a3	-0.1516	0.038747
Zbtb8b	-0.15149	0.027241
Icam5	-0.15137	0.023742
2810453I06Rik	-0.15102	0.031768
Nhs	-0.1507	0.047212
Thsd3	-0.15032	0.046399
Pramel7	-0.15016	0.044338
Zfp689	-0.14997	0.019808
EG620899	-0.14978	0.021945
Cck	-0.14973	0.031675
Bfsp2	-0.14951	0.038984
BC038167	-0.14941	0.036212
Slamf8	-0.14918	0.049948
Gpr142	-0.14902	0.02518
Pou2f2	-0.14802	0.018496
Wnk2	-0.14797	0.01935
Prss29	-0.14794	0.029931
Hydin	-0.14739	0.037273
EG622432	-0.14721	0.046542
Olfr791	-0.14695	0.045341
Rgn	-0.14643	0.029192

Dak	-0.14491	0.047288
5830411N06Rik	-0.14479	0.035918
Nrl	-0.14419	0.037786
Dusp9	-0.14418	0.041152
Hes5	-0.14398	0.027345
EG629678	-0.14387	0.045344
OTTMUSG00000005162	-0.14352	0.028509
Wipf3	-0.14295	0.027382
EG665887	-0.14186	0.046253
Tcea1	-0.14162	0.024619
Dpf1	-0.14142	0.045677
Spinlw1	-0.14136	0.04584
Slitrk3	-0.14058	0.022219
Arhgdig	-0.14054	0.035177
LOC100048615	-0.14043	0.030577
Tex16	-0.1396	0.044328
Rnf186	-0.13857	0.039831
BC043934	-0.13773	0.04458
Col19a1	-0.13695	0.023405
Crisp3	-0.13678	0.041669
Fbxl7	-0.13608	0.047813
Rpap1	-0.13509	0.0289
Zfp358	-0.13493	0.048833
Slc1a6	-0.13428	0.047418
ENSMUSG00000049982	-0.13358	0.029368
Atad4	-0.13325	0.040191
Adamtsl5	-0.13252	0.030539
Arl4c	-0.13226	0.04505
V1rc16	-0.13158	0.037491
Serpinb1c	-0.13072	0.049047
Barhl2	-0.12939	0.037136
Prss32	-0.12618	0.027336
Olfr66	-0.12356	0.04886
Sstr1	-0.11816	0.04312
EG631906	-0.11723	0.038677
V1rh2	-0.11359	0.049585
Lrrc29	-0.11157	0.038377
1200015N20Rik	0.105208	0.04804
Kctd20	0.111652	0.040691
Rars2	0.114474	0.041733
Usp21	0.114958	0.047303
Cnot10	0.115514	0.047523
Lsg1	0.117324	0.047156
Pbx2	0.117646	0.047072
Glg1	0.117736	0.049863
Phc3	0.117804	0.037574
BC059842	0.118284	0.035235
Recql	0.118483	0.044592
Arl3	0.118555	0.045619

Plekhm2	0.119989	0.033429
Fastkd1	0.121105	0.04413
Syne2	0.122441	0.04908
App	0.122855	0.048423
Zfp276	0.122915	0.046392
Sap130	0.12365	0.030179
Mllt1	0.124156	0.043126
Dapk1	0.124513	0.047912
Klhl18	0.124718	0.04767
Pik3ca	0.124719	0.02558
Fastkd5	0.125223	0.030588
Nme1	0.12541	0.041183
1700011F03Rik	0.125413	0.033099
Rint1	0.125734	0.038098
Nxt1	0.125739	0.036926
Zfp294	0.12579	0.04059
Hmg20a	0.125886	0.035696
Them4	0.126169	0.022123
Prrg2	0.126269	0.046693
Shq1	0.126456	0.029047
Qrsl1	0.127063	0.044299
4932442K08Rik	0.127259	0.036141
Hspa9	0.127758	0.037511
Tsen34	0.128146	0.029161
Gmcl1	0.128941	0.035155
Lrrc14	0.129037	0.019641
Dhdds	0.129202	0.039461
Ap1gbp1	0.129365	0.037453
Nucb1	0.13063	0.036566
1500001M20Rik	0.130995	0.044226
Ercc3	0.131045	0.031274
Ccdc95	0.131078	0.043305
Tmod3	0.131546	0.042366
Tsfm	0.131547	0.045148
Gtf3c1	0.131584	0.039774
Kptn	0.131589	0.040549
Elp3	0.131826	0.044968
1700055N04Rik	0.132112	0.039167
Git2	0.13214	0.040988
Upf1	0.132318	0.049411
Thop1	0.133129	0.049438
Nudt16l1	0.13363	0.043024
Pi4kb	0.134159	0.046515
Fbl	0.134202	0.03727
Ccdc100	0.13424	0.049503
1810048J11Rik	0.134806	0.043728
Sh3bp5l	0.134844	0.043399
Centg2	0.134989	0.021942
Atp6v0a2	0.135077	0.03152

Rhod	0.135522	0.028938
Paf1	0.135794	0.028069
Zbtb7b	0.136074	0.048259
Eno1	0.136147	0.036161
D2Ertd391e	0.136286	0.033326
Cln3	0.13651	0.04254
Slc39a9	0.136768	0.049257
Yrdc	0.137142	0.038871
Spna2	0.137577	0.032885
Polr1e	0.13782	0.041291
Mett10d	0.137835	0.042412
Nol1	0.137869	0.024167
Csrp1	0.137982	0.046134
Gabpb2	0.138058	0.049216
Arhgef3	0.138089	0.033982
Sart1	0.13809	0.038761
Rexo2	0.138126	0.044614
Tnpo2	0.138605	0.017683
OTTMUSG00000014597	0.138823	0.04688
Snd1	0.138938	0.047877
Gak	0.139014	0.040527
Cdk5rap3	0.139181	0.047276
LOC626152	0.1392	0.020343
Gnptab	0.139236	0.042174
Nlk	0.139256	0.033774
Cdk4	0.139525	0.044831
2010204K13Rik	0.139802	0.044242
Mid1	0.139804	0.035844
Usp19	0.139812	0.033029
Ttll12	0.139827	0.03694
Arfgef2	0.14061	0.043868
Gfm1	0.141221	0.039332
Ccdc94	0.141817	0.04651
Gdap2	0.141874	0.030173
Frap1	0.141998	0.032449
Ext2	0.142562	0.04967
Ube2f	0.142669	0.043528
Harbi1	0.142808	0.04493
Spg11	0.142907	0.033682
Acot8	0.142999	0.013835
Cacybp	0.143023	0.043232
Tpp1	0.143109	0.031864
Dgcr2	0.143232	0.046134
Pdcd11	0.143309	0.018141
Mis12	0.143392	0.042237
Zfp592	0.144017	0.021354
Wdr18	0.144506	0.031796
Mta1	0.144638	0.025914
Ddx23	0.145099	0.035146

Sart3	0.145131	0.040823
Prcc	0.145448	0.045594
Mrpl12	0.145523	0.041594
Pi4ka	0.14573	0.039049
Cnpy2	0.145985	0.027466
Ccbl1	0.146702	0.048894
Eno1	0.146968	0.027711
Ddx54	0.147261	0.037068
Cldn12	0.147468	0.038897
Afg3l1	0.147709	0.020574
Erlin2	0.147777	0.0274
Ercc4	0.147908	0.041356
Txndc15	0.1484	0.037277
Tgfbrap1	0.148425	0.040823
6430548M08Rik	0.14848	0.042683
Gtpbp1	0.148534	0.024486
Rae1	0.148554	0.041487
Tbl1x	0.14861	0.034635
Cherp	0.148682	0.045564
Sertad3	0.148817	0.030332
Parvg	0.14891	0.039012
Timm44	0.148984	0.012696
Psmf1	0.149019	0.038718
100040879	0.149145	0.030285
C530043G21Rik	0.149188	0.038766
A630047E20Rik	0.149325	0.031199
Pold2	0.14938	0.040878
Zdhhc14	0.14969	0.036583
BC004004	0.149693	0.039818
Tm9sf4	0.149855	0.041851
Hmgcl	0.149949	0.035708
Tmem39b	0.15075	0.046603
Ranbp6	0.150953	0.021311
Clcn7	0.150983	0.042559
Ppan	0.151016	0.04712
Slbp	0.15139	0.031353
Sfmbt1	0.151401	0.037051
Wwc2	0.151619	0.044254
Mrps5	0.151685	0.025912
Pprc1	0.151835	0.049398
Spg7	0.151886	0.02422
Cdc6	0.152054	0.032011
U2af2	0.152239	0.024535
Parl	0.152326	0.041392
3930401K13Rik	0.152436	0.026263
Nat11	0.15256	0.024748
Uap1l1	0.152635	0.042928
Slc10a7	0.152855	0.044949
Sys1	0.152863	0.02729

Phb2	0.15288	0.045827
Heatr6	0.152981	0.021326
Sars	0.153143	0.024215
Noc3l	0.153163	0.047926
Slc7a5	0.153248	0.041601
Ascc3l1	0.153293	0.027426
Taf12	0.153563	0.037847
Eif2ak3	0.153766	0.02107
Cyp2r1	0.15404	0.04991
Prmt2	0.154103	0.045132
Ift172	0.154673	0.039101
Mxd4	0.154737	0.04093
Mrs2	0.155089	0.04074
Golgb1	0.155107	0.031654
BC057893	0.15549	0.036855
Gart	0.155734	0.019435
Rab35	0.155826	0.026213
Prelid2	0.155927	0.047611
Telo2	0.156058	0.032122
Mbtps1	0.156208	0.020969
Pgm1	0.156375	0.018483
Ctdspl	0.156454	0.029635
Pgam1	0.156672	0.042057
Wdsub1	0.156699	0.028709
Syf2	0.156832	0.028794
Prkd3	0.156923	0.015734
Syne2	0.156959	0.015794
Mlx	0.157009	0.043867
Man1b1	0.157035	0.013429
Jagn1	0.157061	0.042839
Ecd	0.157207	0.047009
Gpr180	0.157214	0.021657
Asna1	0.157632	0.04204
Prpf3	0.157746	0.017167
Tars2	0.157983	0.025168
Rragb	0.157986	0.017641
4933426G20Rik	0.158295	0.029172
Dcun1d3	0.159224	0.039988
Dsn1	0.159309	0.04338
Mettl1	0.159758	0.042011
Uprt	0.160212	0.027912
Ruvbl2	0.160239	0.027662
Prep	0.160353	0.049224
Abt1	0.160487	0.043915
EG627967	0.160734	0.030778
Mocs1	0.16096	0.032809
Htf9c	0.161037	0.03535
Ppig	0.161206	0.033893
Alg2	0.161331	0.035439

Pde8a	0.161436	0.032357
Pkp4	0.161474	0.043771
Dguok	0.161588	0.045048
Nudt9	0.161693	0.034849
Mtrr	0.161759	0.03142
Plscr1	0.162493	0.024197
Zc3h13	0.162594	0.028174
Myo1b	0.162867	0.041135
Qtrtd1	0.162958	0.026354
Adck1	0.162972	0.030937
Sap30bp	0.163011	0.048301
Ptrh2	0.163035	0.033961
Bfar	0.163191	0.02658
Letm1	0.163192	0.026863
Nbea	0.163377	0.021442
Tspan3	0.163397	0.03377
Ferd3l	0.163446	0.019131
Fcgr3	0.163462	0.019515
Eya3	0.163521	0.025625
Rfng	0.163548	0.020963
B4galnt1	0.163597	0.046726
Las1l	0.163629	0.039491
Tomm40l	0.163643	0.017323
Copg	0.163662	0.045051
Ptcd1	0.163696	0.045709
Rpp14	0.163881	0.046831
Diablo	0.163957	0.01016
Scamp4	0.164244	0.034631
Pak1ip1	0.16429	0.014029
Cntln	0.164712	0.019014
Dlst	0.165076	0.030032
Man2c1	0.165323	0.034068
Ap1s1	0.165402	0.025535
Ddx41	0.165417	0.030851
Slc5a6	0.165485	0.021729
Mynn	0.165654	0.035678
Lrsam1	0.165741	0.043691
Snx17	0.1658	0.046885
Sec14l2	0.165838	0.033057
Trmt12	0.166112	0.021584
2410089E03Rik	0.166141	0.018635
Edil3	0.166186	0.028447
OTTMUSG00000000657	0.166286	0.030957
Mrps34	0.166422	0.039373
Stx2	0.166612	0.021123
Mtap9	0.166679	0.03819
Ndnl2	0.166844	0.042542
D430028G21Rik	0.166864	0.039686
Tyw1	0.166954	0.027514

Dcps	0.167223	0.030852
Dnase1l3	0.167266	0.022199
Cdk10	0.167456	0.013717
Rpl7l1	0.167863	0.031678
Slc35b1	0.168002	0.043606
Lsm1	0.168058	0.043144
Sf3b4	0.168137	0.015351
Irf3	0.168192	0.027997
Slc41a1	0.168431	0.026539
Rangap1	0.16864	0.046945
Smarcb1	0.169435	0.041601
Zfp187	0.169487	0.049664
Agpat6	0.169571	0.036819
Wbp11	0.169572	0.018009
Ppp1r15b	0.16976	0.028112
Nr1h2	0.169761	0.014771
Alg1	0.170515	0.021831
Tsta3	0.170536	0.032247
Srm	0.170593	0.02691
Stom	0.170639	0.026045
ENSMUSG00000002791	0.1708	0.024111
OTTMUSG00000011448	0.171189	0.028061
Rbm14	0.171321	0.01617
Axl	0.171338	0.023961
Dohh	0.1714	0.04782
1110005A23Rik	0.171516	0.048555
Mrps7	0.171524	0.018134
Cdk10	0.17154	0.027311
Rnps1	0.171767	0.041419
Mall	0.171798	0.015388
Pak4	0.172075	0.011002
Myg1	0.172163	0.009687
D0HXS9928E	0.17224	0.025099
Clp1	0.172279	0.0248
Ccdc124	0.172873	0.029088
Slc35b4	0.173074	0.035356
Tubgcp4	0.173086	0.034285
Rapgef3	0.173114	0.027856
Mepce	0.17344	0.017564
Arpc4	0.173561	0.048851
Cyp4f13	0.173726	0.042788
Ndufa8	0.173852	0.043976
Pdia4	0.173875	0.028064
Gbp1	0.174241	0.035586
Samm50	0.174432	0.028002
Pop4	0.174551	0.037171
Ddx39	0.175007	0.01558
Fen1	0.175231	0.014628
Grwd1	0.175365	0.02675

Pot1a	0.175763	0.008593
Sipa1l1	0.176002	0.032916
Wdr60	0.176285	0.008975
Gars	0.176363	0.028814
Iqcf3	0.176491	0.012639
Cirh1a	0.176685	0.044937
Slc25a32	0.177442	0.025697
Chst12	0.177612	0.042645
Ppt2	0.177748	0.039483
Myl6	0.178049	0.043146
R3hcc1	0.17842	0.035288
Shkbp1	0.178554	0.0249
Bop1	0.178584	0.02047
Grpel1	0.178888	0.008574
Erp29	0.179383	0.040432
Tmem208	0.179422	0.043684
Rpl23	0.179905	0.025503
Chid1	0.180064	0.028734
Entpd6	0.18051	0.043709
St13	0.18056	0.041706
LOC100048603	0.18059	0.026119
Sap30l	0.180774	0.043282
Tut1	0.180873	0.014672
Tbc1d17	0.180873	0.020908
Slc38a7	0.181229	0.010068
Slc39a7	0.181284	0.015453
Adck4	0.181544	0.018258
Txndc5	0.182035	0.045309
Nagk	0.182219	0.029789
Gpr108	0.182229	0.033352
Ascc1	0.182629	0.036007
Nr2f6	0.183035	0.011741
Gpatch1	0.183106	0.007236
Exosc2	0.183309	0.017315
Cbr4	0.183379	0.043625
Mcm2	0.183678	0.029282
Mrpl10	0.183927	0.015367
Zbtb2	0.18428	0.031981
Rpl23	0.184748	0.025368
Gbp4	0.185008	0.019104
Nkap	0.185207	0.047763
Ldhb	0.185397	0.049263
3110040N11Rik	0.185492	0.008709
Myl9	0.186011	0.047443
Tmed9	0.186606	0.012084
AA673488	0.186954	0.006646
Jag1	0.187315	0.009638
Med31	0.187602	0.017537
1190005F20Rik	0.187761	0.014675

Sema4b	0.188019	0.021609
Adcy3	0.188038	0.003842
Prpf31	0.188154	0.02608
2510003E04Rik	0.188165	0.034753
Cope	0.188216	0.043806
Ncbp2	0.188538	0.020158
Dnaja3	0.18868	0.015687
Nrbp2	0.189344	0.025754
Phb	0.189654	0.019305
Mrps33	0.189903	0.036163
Creld2	0.189923	0.036598
Psmb10	0.190089	0.02757
Farsa	0.190113	0.02121
L3mbtl2	0.190608	0.022813
Zfp598	0.190634	0.007162
Slc19a1	0.190787	0.032634
Afap1	0.190852	0.046272
Pira3	0.191264	0.029933
Rrp1	0.191278	0.011801
Srprb	0.191573	0.013897
1810032O08Rik	0.191622	0.030746
Vac14	0.191673	0.036986
Pop5	0.19216	0.00899
Bag4	0.192436	0.027739
Stard8	0.19257	0.028681
Ggcx	0.193989	0.02121
Tbcb	0.194172	0.036798
Fancm	0.194972	0.02813
Terf2	0.195002	0.005099
Rai12	0.195155	0.026523
Gm1040	0.195402	0.020261
Shb	0.195844	0.021164
Tufm	0.195881	0.017073
Ppp4c	0.19673	0.034257
Aarsd1	0.196744	0.023321
Flot2	0.197162	0.040653
Usp5	0.197391	0.048784
Prmt6	0.197502	0.008074
Suclg1	0.197601	0.039126
Tmed3	0.197646	0.027516
Coq6	0.197881	0.037213
Lgi4	0.198667	0.024689
Prkcsh	0.198773	0.034519
Cotl1	0.199348	0.038495
Lrrc8c	0.199537	0.040733
Frrs1	0.200056	0.018751
5830443L24Rik	0.200302	0.036475
Faim	0.20038	0.043968
Upf3a	0.200386	0.007606
Dnpep	0.200513	0.021256

Ogfod1	0.201113	0.00799
0610031J06Rik	0.201319	0.047228
Mafg	0.201418	0.025585
Ssr2	0.202124	0.025697
Rpusd4	0.202474	0.022083
Cyp2j6	0.202597	0.047677
Hyou1	0.202612	0.045371
Il10	0.202723	0.021215
Phb	0.202861	0.022167
Aldh18a1	0.203108	0.016617
Imp4	0.20323	0.03771
Rad50	0.203656	0.00771
Syne2	0.203666	0.002704
Mrps33	0.203887	0.03079
2310011J03Rik	0.20411	0.014979
Nudt22	0.204336	0.033481
Angptl3	0.205283	0.042336
Oprs1	0.20552	0.028432
Lama4	0.205781	0.048876
Hadha	0.206074	0.046703
Prkaca	0.206075	0.035234
Taf5	0.206122	0.011102
Crip2	0.206462	0.043496
1110049F12Rik	0.207157	0.038472
Sf3a1	0.207168	0.019473
Wars	0.207391	0.013553
Tufm	0.207917	0.00845
4930504E06Rik	0.207996	0.007314
Hoxc8	0.208493	0.022119
Usp49	0.208552	0.016967
Pecr	0.209243	0.026803
9030617O03Rik	0.21009	0.031575
Mmaa	0.210159	0.027865
Slc39a10	0.210292	0.022427
Gtf2e1	0.210478	0.02468
Mtmr9	0.210951	0.013508
Capg	0.210993	0.012135
Gss	0.21106	0.047225
Qdpr	0.211114	0.028929
Crtap	0.211444	0.036781
Tagln	0.211552	0.037706
Npr2	0.211656	0.039991
Pla2g2d	0.21222	0.028729
Zfat	0.213478	0.008062
Ctnnbl1	0.213904	0.021964
Smpd1	0.214137	0.026484
Cgnl1	0.214221	0.033916
Lman2	0.215493	0.019381
Plod3	0.21662	0.010742

Kank3	0.216853	0.026816
Xpnpep3	0.217114	0.040996
Ddhd2	0.217159	0.048344
666761	0.217169	0.022746
Slc26a4	0.217243	0.007917
Katnal1	0.218036	0.010587
Psat1	0.218865	0.02825
N6amt2	0.218876	0.048091
Dus2l	0.219671	0.027533
A230067G21Rik	0.221068	0.005492
Crls1	0.221202	0.0458
Cyyr1	0.221216	0.035881
Mcm3	0.221294	0.02291
Spnb2	0.22147	0.033885
Taf11	0.222148	0.00541
Nat9	0.223007	0.012913
Gstm4	0.223024	0.039692
Gsto2	0.223216	0.036548
Zfp294	0.223403	0.006569
Prdm9	0.223682	0.010702
Mrpl22	0.22417	0.041541
Tubb2b	0.224257	0.028309
Phlda3	0.224282	0.039321
Slc22a5	0.225063	0.022274
Dock6	0.225138	0.013651
Itpk1	0.225147	0.048174
Zc3h12a	0.225451	0.007051
Cul7	0.225461	0.013604
Rnpep	0.225708	0.028374
Mut	0.225863	0.046398
Hsdl2	0.226774	0.045452
Gnb5	0.227771	0.016616
4932441K18Rik	0.229172	0.044844
Wrb	0.22968	0.008522
Bphl	0.229775	0.038775
Cth	0.23	0.014211
1500003O03Rik	0.23005	0.033848
ENSMUSG00000071392	0.231512	0.005138
Mtus1	0.233195	0.005718
Igfbp4	0.23433	0.046121
Cry2	0.234594	0.027204
Taldo1	0.23501	0.03101
Pomt1	0.235077	0.035157
Alas1	0.235864	0.026406
Cspp1	0.236014	0.009682
Clpb	0.237032	0.018523
Slc10a6	0.23715	0.036713
Nme7	0.238413	0.011987
Cxx1b	0.239926	0.04381

Mertk	0.241155	0.043101
Hrsp12	0.241477	0.04444
Fkbp2	0.242594	0.016428
Apex2	0.243413	0.008526
Has3	0.243589	0.013628
Pmm1	0.243678	0.011723
Ttpa	0.245695	0.007304
Creg1	0.246031	0.031509
Ttc27	0.246698	0.002841
Ablim3	0.247028	0.004043
Casp4	0.247236	0.019611
Xdh	0.247535	0.030437
Coasy	0.247776	0.024888
Ear1	0.247997	0.023517
Akr1e1	0.24816	0.009973
Drg2	0.248449	0.013033
Adra1a	0.248569	0.018977
Ampd2	0.248809	0.006669
Ppa1	0.249021	0.025931
Pcdhb22	0.250766	0.023919
Triap1	0.251814	0.014319
Pex5	0.251816	0.023299
Rpl13	0.252796	0.04254
D2hgdh	0.252819	0.015113
Sprr2j	0.253216	0.034419
Rpp40	0.253663	0.025597
Mtg1	0.255209	0.017277
Yipf3	0.25774	0.008746
Rnf5	0.259822	0.03606
Ptprg	0.260072	0.008184
Eral1	0.260981	0.006463
2310061C15Rik	0.261279	0.020172
Abhd5	0.262318	0.036199
Tmeff1	0.263592	0.04673
Atp10d	0.26418	0.008021
Degs2	0.264851	0.027302
Acadsb	0.265701	0.03014
Uchl5ip	0.267318	0.017758
Lonp1	0.267888	0.002086
Srpx	0.268348	0.024573
Pygl	0.268452	0.008866
Polr2i	0.269423	0.012731
Hint2	0.273091	0.014557
Agpat3	0.273309	0.04483
Snord35b	0.273385	0.03712
Tlr8	0.274775	0.049221
Cbr3	0.27629	0.018108
Fancl	0.277242	0.000845
Hsd17b4	0.279452	0.029691

Cyp4b1	0.280836	0.030512
Ptplb	0.281459	0.014965
Irak1bp1	0.283615	0.033624
Sntb1	0.283697	0.047064
Snrk	0.28382	0.00163
Trap1	0.286188	0.026225
Zfp637	0.286492	0.01544
Scarb1	0.286877	0.029164
Pold4	0.287706	0.043349
Atp9a	0.288252	0.036294
Gulp1	0.288254	0.000319
Ak2	0.291075	0.016629
4932441K18Rik	0.291305	0.01574
1810046J19Rik	0.292891	0.002116
Mccc2	0.293173	0.017009
Entpd5	0.294107	0.024939
Anxa3	0.295564	0.031017
Asns	0.295883	0.030919
Insr	0.296112	0.041332
Adamts9	0.296884	0.037056
Csad	0.297939	0.0257
G6pdx	0.298027	0.017837
Iltifb	0.300343	0.002404
Acaa2	0.300516	0.042505
F10	0.303798	0.048093
Mfsd8	0.304012	0.03293
Gnpat	0.306033	0.046253
Tspan4	0.306507	0.01808
Pp11r	0.308659	0.040583
Hadh	0.310518	0.043749
H6pd	0.316618	0.036383
Ntrk2	0.317723	0.044723
Comt	0.318351	0.041044
Leprel1	0.320726	0.008414
Palmd	0.322173	0.024766
2310042E22Rik	0.322243	0.026161
Steap4	0.323654	0.046589
Slc9a6	0.330506	0.047944
Tenc1	0.331184	0.037679
Bcar3	0.359238	0.045436
Mecr	0.360201	0.014395
Gbp2	0.363605	0.028334
Bscl2	0.365325	0.011735
Pex11a	0.379716	0.024288
Mgll	0.387841	0.038459
1100001G20Rik	0.390513	0.021786
Trf	0.390788	0.024652
Ppp2r1b	0.391846	0.032701
Pfkl	0.396348	0.00208

Gnai1	0.396788	0.016571
Ehhadh	0.400019	0.047034
Cpt2	0.412258	0.034615
EG626785	0.440782	0.001434
Gstt2	0.448686	0.045386
Id3	0.457744	0.024473
Sfxn1	0.459008	0.028413
Asah3l	0.47918	0.001688
Acaca	0.48718	0.025686
Acad11	0.500459	0.024214
Sfrp4	0.514222	0.035332
Mgl2	0.535517	0.018523
Dlc1	0.554076	0.044425
Slc1a3	0.560558	0.028196
Chi3l3	0.58156	0.022181
Aspa	0.584456	0.035218
Abca8a	0.607574	0.046606
Rbp7	0.692221	0.048274
Sdpr	0.737569	0.045736
Acsl1	0.740428	0.036117
Lipe	0.770016	0.042404
D430015B01Rik	0.795781	0.034967
Slc36a2	0.842079	0.046337
Retsat	0.899542	0.037957
Sycp3	0.909188	0.047779
Ucp1	1.118152	0.038291
Pck1	1.537555	0.033318
